# Supplementary figures and images for: Immobilization of Silver Nanoparticles with Defensive Gum of Moringa oleifera for Antibacterial Efficacy Against Resistant Bacterial Species from Human Infections
Source: Pharmaceuticals (Basel). 2024 Nov 18;17(11):1546. doi: 10.3390/ph17111546 (PMC11597128; doi:10.3390/ph17111546)

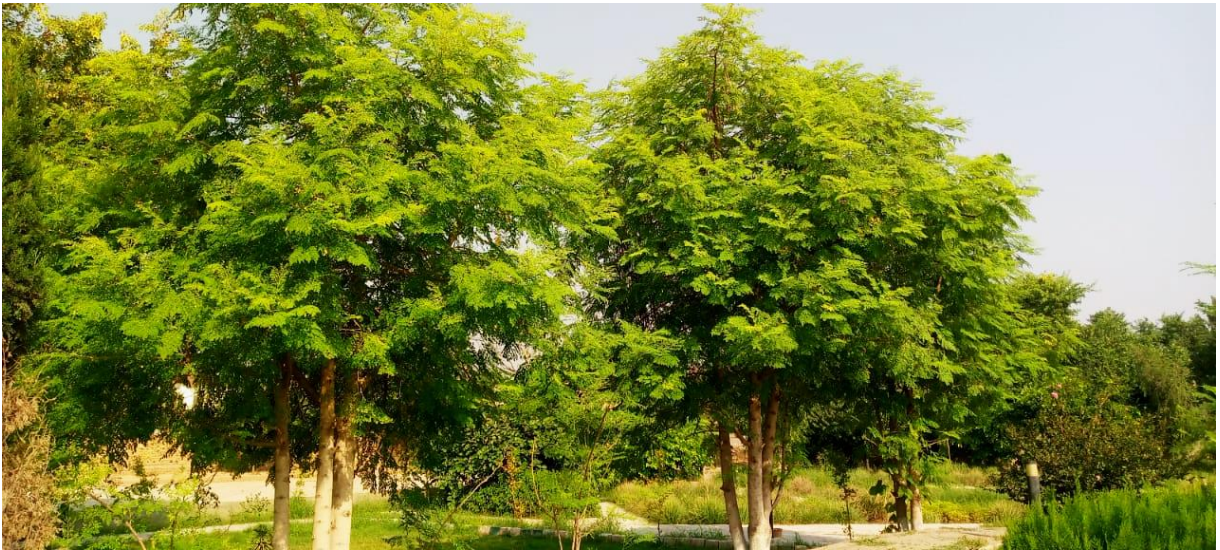

Moringa trees

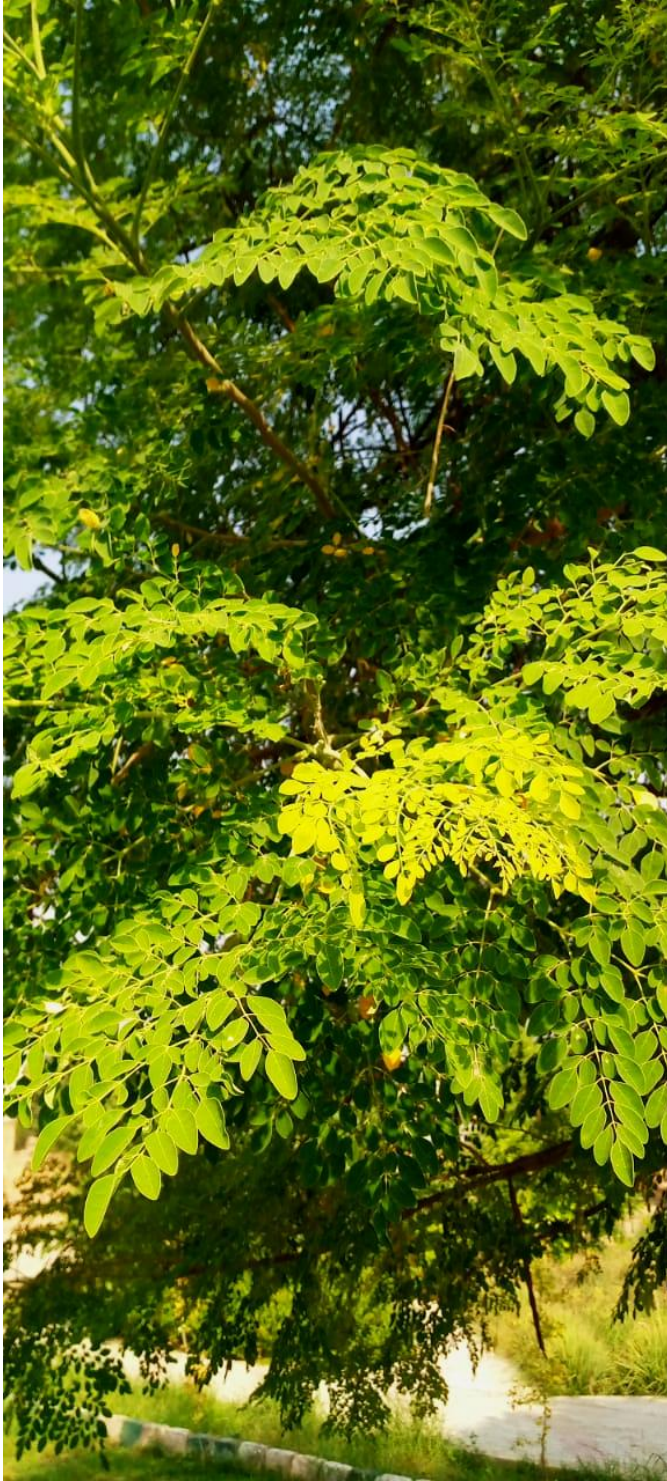

Leaves collected from Moringa tree in Spring season

Supplement: Supplementary file 1 [file pharmaceuticals-17-01546-s001.zip › pharmaceuticals-3228584-supplementary.pdf]
